# Morphometrics of *Amblyomma mixtum* in the State of Veracruz, Mexico

**DOI:** 10.3390/pathogens10050533

**Published:** 2021-04-29

**Authors:** Mariel Aguilar-Domínguez, Dora Romero-Salas, Sokani Sánchez-Montes, Ricardo Serna-Lagunes, Greta Rosas-Saito, Anabel Cruz-Romero, Adalberto A. Pérez de León

**Affiliations:** 1 Laboratorio de Parasitología, rancho “Torreón del Molino”, Facultad de Medicina Veterinaria y Zootecnia, Universidad Veracruzana, Veracruz 91697, Mexico; marieaguilar@uv.mx (M.A.-D.); anabcruz@uv.mx (A.C.-R.); 2 Facultad de Ciencias Biológicas y Agropecuarias Región Tuxpan, Universidad Veracruzana, Tuxpam 92870, Mexico; danisanchez@uv.mx; 3 Laboratorio de Bioinformática y Bioestadística, Facultad de Ciencias Biológicas y Agropecuarias, Universidad Veracruzana, Córdoba 94945, Mexico; rserna@uv.mx; 4 Red de Estudios Moleculares Avanzados, Instituto de Ecología, Xalapa 91073, Mexico; greta.rosas@inecol.mx; 5 USDA-ARS Knipling-Bushland U.S. Veterinary Pest Genomics Center and Livestock Insects Research Laboratory, Kerrville, TX 78028, USA; beto.perezdeleon@usda.gov; 6 USDA-ARS San Joaquin Valley Agricultural Sciences Center, Parlier, CA 93648, USA

**Keywords:** *Amblyomma mixtum*, tick, morphometrics, cattle, Mexico

## Abstract

The tick *Amblyomma mixtum* is an ectoparasite of veterinary and public health importance because of its role as a vector of zoonotic pathogens. However, little is known about *A. mixtum* intraspecific variability and if morphological differentiation exists between populations across its geographic range. This study aimed to determine by electron microscopy the morphological variability of *A. mixtum* populations in the state of Veracruz, which has a large livestock population among states in Mexico. Forty male and 40 female *A. mixtum* collected from the 10 natural regions of Veracruz state were analyzed microscopically to accomplish main component analysis for each sex. Clusters were calculated with the out-tree method and a dendrogram produced to group the specimens according to their morphometric characteristics. Using 10 main components, 77% of the morphological variation of the ticks was explained. This is a reflection of scarce intraspecific variation between females. The dendogram for females grouped six clusters of specimens with similar characteristics. Morphometric variability in males was described using eight main components. Limited intraspecific variation was also observed between males. In males, the dendogram yielded six groups with similar morphometric characteristics. Morphometric analyses confirmed that the only species from the *Amblyomma cajennense* complex that are parasites to livestock in Veracruz state is *A. mixtum*. The eryxenous nature of *A. mixtum* combined with the frequent movement of livestock hosts may contribute to the apparent homogeneous phenotype of this tick species in Mexico.

## 1. Introduction

Ticks are economically important ectoparasites of livestock [1]. Their economic importance derives from the obligate blood feeding habit and role as vectors of pathogens causing significant morbidity and mortality in host populations [2]. Among the approximately 130 extant tick species in the genus *Amblyomma* worldwide, several impact livestock health and production as ectoparasitic disease vectors [3,4]. Nearly half of the known *Amblyomma* species exist in the Americas and in some cases their geographic range is expanding [5,6].

*Amblyomma mixtum* is a valid species in the *A. cajennense* species complex and is considered one of the most economically important tick species parasitizing livestock in Mexico [7,8]. Its impact on livestock health is amplified by the role of *A. mixtum* as a mechanical vector of *Anasplasma* that causes bovine anaplasmosis, which can result in significant mortality among cattle herds, it also parasitizes horses, being an important vector of various species of rickettsiae [9]. Moreover, the public health importance of *A. mixtum* stems from its host range that includes humans, and ability to vector zoonotic pathogens [10]. As it is the case with other *Amblyomma* species in Mexico [11], there are biological aspects of *A. mixtum* that remain to be defined.

Veracruz is a state in Mexico with a diverse livestock population and the largest cattle herd comprising approximately 11% of the total national inventory [12,13]. Syntethic acaricides are used to control ticks infesting livestock including *A. mixtum*. The indiscriminate use of acaricides is a major driver for the emergence of acaricide resistance among populations of *A. mixtum* infesting livestock in Veracruz state [14]. This is concerning because evidence for the emergence of resistance to multiple classes of acaricides is in this three-host tick species even when gene flow associated with lack of genetic differentiation was documented for *A. mixtum* populations in the same region [15]. However, studies to determine if morphological variation among *A. mixtum* population in Veracruz state remained to be conducted.

Morphological anomalies associated with exposure to acaricides was reported for *Amblyomma lepidum* infesting cattle in Uganda [16]. This prompted morphological analysis of our tick collection to determine if a similar situation occurred in *A. mixtum*. Here, we report the morphometrics of *A. mixtum* collected from infested livestock and the surrounding agroecosystems representing the ten ecological regions in the state of Veracruz, Mexico.

## 2. Results

Based on the results of the descriptive statistics of the morphological characters evaluated, it is observed that the males surpassed the females in terms of the dimensions of the CB, TL, LSII, HL, HW and Tal characters, in the remaining morphometric characters, both males and females did not show evident morphological differentiation (Table 1).

**Figure 1:**
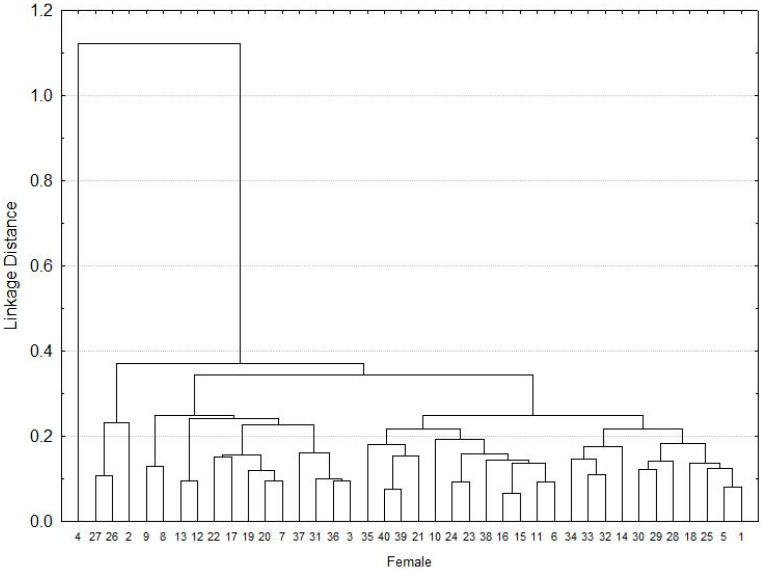
Cluster of the morphometry for *A. mixtum* females.

Based on the morphometric measurements, the females were grouped into three groups, the first made up of three individuals, the second by 12 and the last group was made up of a greater number of females that were more homogeneous morphologically (Figure 1).

**Figure 2:**
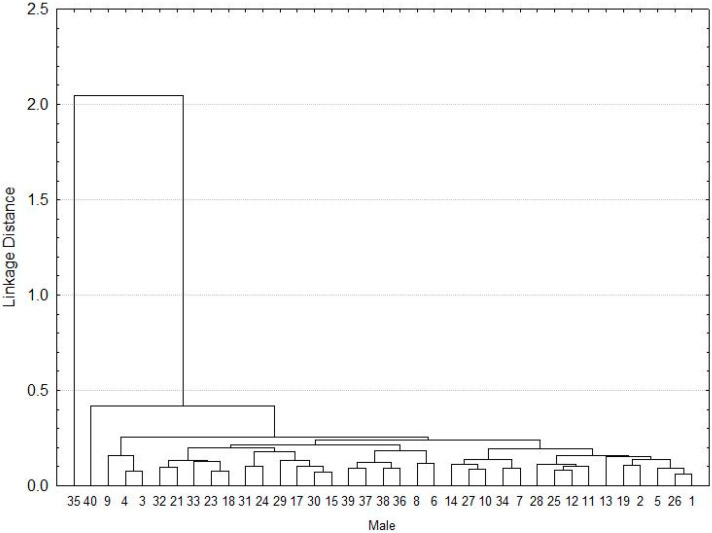
Cluster of the morphometry for *A. mixtum* males.

Regarding the males, two individuals (35 and 40 individual) did not show similarities in the morphometric characteristics of the group made up of the rest (*n* = 38 individuals), which were grouped by the similarity of its morphometric features (Figure 2).

## 3. Discussion

This is the first study to interrogate *A. mixtum* intraspecific variability and morphological differentiation between populations in Mexico. Results of the morphometric analyses confirmed that the only species from the *A. cajennense* complex parasiting cattle in Veracruz state, Mexico is *A. mixtum*. This is in agreement with results from previous studies on the reinstatement of *A. mixtum* as a valid species and the documentation of its geographic range [7,15,17]. It is important to understand the actual distribution of *A. mixtum* because of its relevance in veterinary medicine and public health as an ectoparasite and vector of tick-borne pathogens [6,18].

Evidence recognizing *A. mixtum* as an important ectoparasitic disease vector of vertebrate hosts in Veracruz state continues to accumulate. This tick species was formerly referred to as *A. cajennense* [11,19,20]. The genetic plasticity of *A. mixtum* is reflected in its adaptation to infest the water buffalo [12], which is a livestock species introduced to Mexico [21]. Resistance to multiple classes of acaricides among populations of *A. mixtum* infesting livestock in Veracruz state was confirmed [14].

The *A. mixtum* analyzed in this study were sampled in parts of Veracruz state where livestock are treated intensely with acaricides [14,22].

However, we did not detect morphological anomalies associated with exposure to acaricides as it was reported for *Amblyomma lepidum* infesting cattle in Uganda [16]. Morphological abnormalities have been reported in several *Amblyomma* species [5]. Their detection in field-collected *A. mixtum* likely requires a larger samples collection because morphological anomalies apparently rarely occur [16].

Intraspecific morphological variation was not detected in the male and female *A. mixtum* analyzed in this study. This seemingly homogenous phenotype may reflect the lack of genetic separation between *A. mixtum* populations across the natural regions of Veracruz state [15]. Although it can inhabit diverse ecosystems in Mexico parasitizing multiple vertebrate host species [8,23,24], similar morphometric characteristics were detected in adult *A. mixtum*. The eryxenous nature of *A. mixtum* combined with the frequent movement of livestock hosts may contribute to the apparent homogeneous phenotype of this tick species in Veracruz state, Mexico.

## 4. Materials and Methods

### 4.1. Study Area

The study was carried out in the state of Veracruz; it is located in eastern Mexico and extends along the coastal plain between the Sierra Madre Oriental and the Gulf of Mexico covering an area of 72,410 km^2^. Although located in the tropics, the state encompasses these diverse climates given its varied geography [25].

*Warm humid and subhumid climate.* It comprises a larger area, approximately 80% of the territory of Veracruz, which is distributed in the coastal plains of the North Gulf and South Gulf, at a maximum height of 1000 m a.s.l. The average annual temperature is 22–26 °C.

*Humid semi-warm climate:* In places with an average elevation of 1000 to 1600 m a.s.l., the physical characteristics favor the development of semi-warm climates. The average temperature varies between 18 and 22 °C.

*Temperate climate:* It is registered in the zones with altitudes between 1600 and 2800 m a.s.l.

The ten natural regions are: Huasteca Alta, Huasteca Baja, Totonaca, Nautla, Capital, Sotavento, Mountains, Papaloapan, Tuxtlas, and Olmeca [26].

### 4.2. Sampling

**Figure 3:**
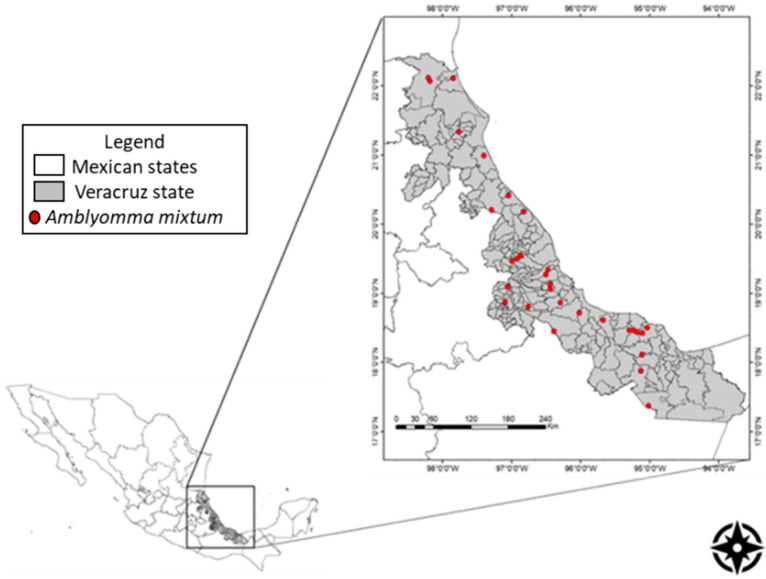
Map of Veracruz state in Mexico with the location of livestock production units within municipalities representing the ten natural state regions where ticks where collected.

Ticks were sampled between August 2014 and January 2015. Tick samples were collected at livestock production units in municipalities encompassing the ten natural state regions (Figure 3).

Livestock production units where the ticks were sampled were located in these municipalities: Pánuco (N 22°4′18.664″, W 98°10′56.038″), Tampico Alto (N 22°6′53.392″, W 97°50′36.963″), Tuxpam (N 20°59′27.391″, W 97°23′47.993″), Naranjos de Amatlán, (N 21°20′8.102″, W 97°45′47.959″), Gutiérrez Zamora (N 20°24′47.999″, W 97°2′30.998″), Papantla (N 20°12′15.998″, W 97°17′15.997″), Nautla (N 20°10′54.984″, W 96°49′26.997″), Xalapa (N 19°29′55.997″, W 96°55′9.998″), Coatepec (N 19°27′42.998″, W 96°59′16.998″), Yanga (N 18°48′9.18″, W 96°45′32.997″), Orizaba (N 18°52′16″, W 97°5′22.999″), Coscomatepec (N 19°5′19″W 97°3′10.998″), Puente Nacional (N 19°20′34.998″, O 96°28′6.999″), Medellín (N 18°51′56.999″, W 96°17′24.997″), Soledad de Doblado, (N 19°3′46.998″, W 96°26′7.997″), Paso de Ovejas (N 19°16′23.999″, W 96°30′5.997″), Tlacotalpan, (N 18°36′53.197″, W 95°40′13.727″), Tierra Blanca (N 18°27′0.288″, W 96°22′54.004″), Ignacio de la Llave (N 18°43′5.045″, W 96°0′52.927″), San Andrés (N 18°27′39.55″, W 95°13′19.462″), Catemaco (N 18°30′5.256″, W 95°1′51.65″), Santiago Tuxtla (N 18°28′3.562″, W 95°17′31.855″), Acayucan (N 18°6′43.218″, W 95°6′24.004″), San Juan Evangelista (N 17°52′31.267″, W 95°7′26.936″) and Jesús Carranza (N 17°22′1.852″, W 95°0′37.908″).

### 4.3. Tick Collection from Infested Livestock

Cattle and horses at the livestock production units were inspected visually and manually from head to tail to ascertain tick infestation. When detected, ticks were detached by gentle traction movement of the fingers to avoid rupture of the gnathosoma. Tick samples were stored in a vial correctly coded that contained 70% ethanol. Vials with the tick samples were taken to the Laboratory of Parasitology in the Torreon del Molino Diagnostic Unit of the Veracruz State University College of Veterinary Medicine in Veracruz, Veracruz. Table 2 lists the sex and number of *A. mixtum* adult ticks collected from livestock hosts sampled at different municipalities in Veracruz state, Mexico.

### 4.4. Morphological Identification of Ticks

Ticks sampled were placed on a slide with the help of small tweezers. Morphological characteristics were observed using a Motic^®^ stereoscope microscope with a camera Moticam 1000 (Speed Fair, Hong Kong, China). Ticks were identified using morphological keys [11,27,28]. Males and females were counted and separated for further processing. Table 2 summarizes the sex and number of *A. mixtum* adult ticks collected from livestock hosts at the different municipalities sampled.

### 4.5. Scanning Electron Microscopic Analysis

Ticks identified as *A. mixtum* were inspected for quality control to ensure processing for scanning electron microscopy. Two female and two male *A. mixtum* representing each state region were processed to obtain dorsal and ventral views. Tick samples were cleaned using extra fine Dumont^®^ Antimagnetic-E tweezers and micro spatulas. Once all the dirty material was removed from the ticks, they were cleaned with a micro brush number 5. Each tick was stored in a white bake lite cap glass vial with 70% ethanol.

Sample downstream processing was done according to Dixon et al. (2000) with some modifications. Briefly, ticks previously stored in 70% ethanol underwent two ultrasonic cleanings for 5 min each using a Cole-Parmer^®^ 8848 ultrasonic cleaner. For dehydration, the 70% ethanol was replaced with 90% ethanol. After 90 min in 90% ethanol, ticks were placed in 100% ethanol three times for 30 min each. Thereafter, the ticks were placed in xylene and maintained at a temperature of 40 °C for 24 h. Then, they were subjected to three washes of 30 min each with absolute ethanol, which was followed by critical point drying with a dryer Quorum^®^ model K850 where the ethanol was exchanged for CO_2_. Once the ticks were completely dry and ethanol free, they were placed in the desired position on a polished aluminum stub covered by a conductive carbon adhesive tape. All the ticks were then coated with gold by the “sputter coating” method using the Coater Quorum^®^ model Q150R S. All the samples were analyzed using the scanning electron microscope FEI Quanta 250 FEG (Oregon, USA).

### 4.6. Morphometrics

**Figure 4:**
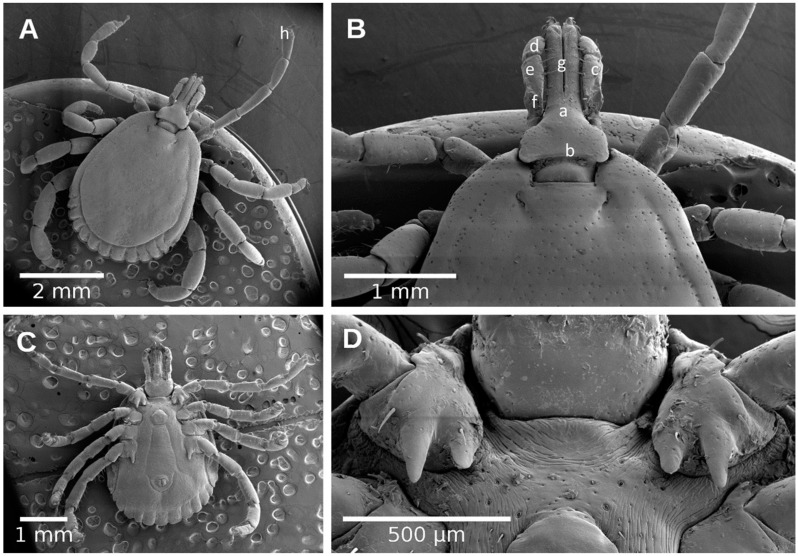
Male of *Amblyomma mixtum*. (**A**) Dorsal view; (**B**) Dorsal view of capitulum; (**C**) Ventral view; (**D**) Ventral view of capitulum. (a) capitulum; (b) basis capitulum; (c) palpi; (d) palpi segment I; (e) palpi segment II; (f) palpi segment III; (g) hypostome; (h) tarsi.

**Figure 5:**
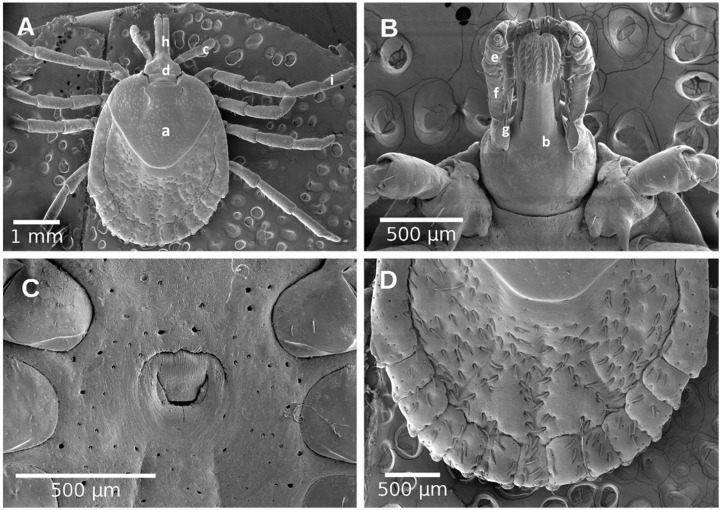
Female of *Amblyomma mixtum*. (**A**) Dorsal view; (**B**) Ventral view of capitulum; (**C**) Genital aperture; (**D**) fentoons; (a) scutum; (b) capitulum; (c) basis capitulum; (d) palpi; (e) palpi segment I; (f) palpi segment II; (g) palpi segment III; (h) hypostome; (i) tarsi.

Morphometric relationships were evaluated separately for males (Figure 2) and females (Figure 4 and Figure 5). Sixteen morphological variables were analyzed for females and 14 for males, where we do not include the scutum (letters of the alphabet associated with the principal components in Table 1). These included: 1) scutum length (SL), 2) scutum width (SW), 3) dorsal capitulum breadth (CB), 4) dorsal capitulum length (CL), 5) basis capituli ventral length (VL), 6) palpi total length (TL), 7) length segment I (LSI), 8) length segment II (LSII), 9) length segment III (LSIII), 10) width segment I (WSI), 11) width segment II (WSII), 12) width segment III (WSIII), 13) hypostome length (HL), 14) hypostome width (HW), 15) tarsi length (TL), 16) tarsi width (TW).

Arithmetic Averages (UPGMA) clustering was used to perform the Unweighted Pair Group Method. Results were displayed through Principal Component Analysis (PCA). PCA was used to further define the clusters, and to evaluate the contributions of individual characters to the phenetic differences among clusters. Eigenvectors were extracted from a pair-wise matrix of Pearson product–moment correlations of the characters. A phenogram (UPGMA) was constructed using the distance matrix. All statistical analyses were done using SAS 9.0.

## Figures and Tables

**Table 1:** Descriptive statistics (mean and standard deviation) of the morphometry of *A. mixtum* by sex category, scutum length (SL), scutum width (SW), dorsal capitulum breadth (CB), dorsal capitulum length (CL), basis capituli ventral length (VL), palpi total length (TL), length segment I (LSI), length segment II (LSII), length segment III (LSIII), width segment I (WSI), width segment II (WSII), width segment III (WSIII), hypostome length (HL), hypostome width (HW), tarsi length (TaL), tarsi width (TW). The average values and their standard deviation obtained from the components that analyze the morphometric variation of *A. mixtum* are shown.

Sex	Statistic	CB	CL	VL	TL	LSI	LSII	LSIII	WSI	WSII	WSIII	HL	HW	TaL	TW	SL	SW
♀	Mean	0.5	0.4	0.3	0.7	0.1	0.5	0.2	0.1	0.1	0.2	0.7	0.2	0.7	0.2		
	SD	0.04	0.03	0.03	0.1	0.01	0.04	0.03	0.01	0.01	0.02	0.03	0.01	0.03	0.01		
♂	Mean	0.7	0.4	0.3	0.9	0.1	0.6	0.2	0.1	0.1	0.2	0.9	0.3	0.9	0.2	1.9	1.9
	SD	0.02	0.02	0.02	0.03	0.02	0.02	0.02	0.01	0.01	0.02	0.03	0.02	0.04	0.03	0.2	0.1

**Table 2:** Sex and number of *A. mixtum* adult ticks collected from livestock hosts sampled at different municipalities in Veracruz state, Mexico.

Number of Ticks		Livestock Host	Municipality
♂	♀		
2	2	*Bos taurus*	Pánuco
1	2	*Equus caballus*	Pánuco
1		*Equus caballus*	Tampico Alto
2	2	*Bos taurus*	Tuxpam
2	2	*Equus caballus*	Naranjos Amatlán
2	2	*Bos taurus*	Gutiérrez Zamora
2	1	*Equus caballus*	Gutiérrez Zamora
	1	*Equus caballus*	Papantla
2	2	*Bos taurus*	Nautla
2	2	*Equus caballus*	Nautla
2	2	*Bos taurus*	Xalapa
2	1	*Equus caballus*	Xalapa
	1	*Equus caballus*	Coatepec
2		*Bos taurus*	Yanga
	1	*Equus caballus*	Yanga
	1	*Bos taurus*	Orizaba
2	1	*Equus caballus*	Coscomatepec
	1	*Bos taurus*	Coscomatepec
1	1	*Bos taurus*	Puente Nacional
	1	*Equus caballus*	Puente Nacional
1		*Bos taurus*	Medellín
	1	*Bos taurus*	Soledad de Doblado
1		*Equus caballus*	Soledad de Doblado
	1	*Equus caballus*	Paso de Ovejas
2		*Bos taurus*	Tlacotalpan
1	1	*Equus caballus*	Tlacotalpan
	1	*Bos taurus*	Tierra Blanca
	1	*Equus caballus*	Tierra Blanca
	1	*Bos taurus*	Ignacio de la Llave
1		*Equus caballus*	Ignacio de la Llave
1	2	*Bos taurus*	San Andrés
1		*Equus caballus*	San Andrés
1		*Bos taurus*	Catemaco
	2	*Equus caballus*	Catemaco
1		*Equus caballus*	Santiago Tuxtla
1	1	*Bos taurus*	Acayucan
1	1	*Equus caballus*	Acayucan
1	1	*Bos taurus*	San Juan Evangelista
1		*Equus caballus*	San Juan Evangelista
	1	*Equus caballus*	Jesús Carranza
